# Loss of *Mecp2* Causes Atypical Synaptic and Molecular Plasticity of Parvalbumin-Expressing Interneurons Reflecting Rett Syndrome–Like Sensorimotor Defects

**DOI:** 10.1523/ENEURO.0086-18.2018

**Published:** 2018-09-24

**Authors:** Noemi Morello, Riccardo Schina, Federica Pilotto, Mary Phillips, Riccardo Melani, Ornella Plicato, Tommaso Pizzorusso, Lucas Pozzo-Miller, Maurizio Giustetto

**Affiliations:** 1 Department of Neuroscience, University of Turin, Corso M. D’Azeglio 52, Turin, 10126, Italy; 2 Department of Neurobiology, University of Alabama at Birmingham, Birmingham, AL 35294, USA; 3 Department of Neuroscience, Psychology, Drug Research and Child Health NEUROFARBA, University of Florence, Area San Salvi Pad. 26, Florence, 50135, Italy; 4 Institute of Neuroscience, National Research Council (CNR), via Moruzzi 1, Pisa, 56124, Italy; 5 National Institute of Neuroscience-Italy, Corso M. D’Azeglio 52, Turin, 10126, Italy

**Keywords:** Cerebral cortex, neuroanatomy, parvalbumin-expressing interneurons, Rett syndrome, structural synaptic plasticity, X-linked intellectual disability

## Abstract

Rett syndrome (RTT) is caused in most cases by loss-of-function mutations in the X-linked gene encoding methyl CpG-binding protein 2 (*MECP2*). Understanding the pathological processes impacting sensory-motor control represents a major challenge for clinical management of individuals affected by RTT, but the underlying molecular and neuronal modifications remain unclear. We find that symptomatic male *Mecp2* knockout (KO) mice show atypically elevated parvalbumin (PV) expression in both somatosensory (S1) and motor (M1) cortices together with excessive excitatory inputs converging onto PV-expressing interneurons (INs). In accordance, high-speed voltage-sensitive dye imaging shows reduced amplitude and spatial spread of synaptically induced neuronal depolarizations in S1 of *Mecp2* KO mice. Moreover, motor learning-dependent changes of PV expression and structural synaptic plasticity typically occurring on PV^+^ INs in M1 are impaired in symptomatic *Mecp2* KO mice. Finally, we find similar abnormalities of PV networks plasticity in symptomatic female *Mecp2* heterozygous mice. These results indicate that in *Mecp2* mutant mice the configuration of PV^+^ INs network is shifted toward an atypical plasticity state in relevant cortical areas compatible with the sensory-motor dysfunctions characteristics of RTT.

## Significance Statement

Understanding the pathologic processes impacting somatosensory processing and motor control represents a major challenge for clinical management of individuals affected by Rett syndrome. We found that *Mecp2* is important, starting from a young age, for the correct molecular and synaptic organization of parvalbumin-positive interneurons in cortical areas responsible for sensory-motor skills. Intriguingly, even partial *Mecp2* loss produces an atypical upregulation of parvalbumin expression in these cells that correlates with the severity of motor behavioral impairments and is associated with defective cortical activity in KO mice. Because in behaviorally impaired *Mecp2* KO mice excessive activity-dependent excitatory connectivity is established with these interneurons, our study suggests that partial inhibition of parvalbumin-positive cells would be beneficial for motor impairments in Rett syndrome.

## Introduction

Classic Rett syndrome (RTT) is a childhood neurologic disorder affecting ∼1 in 10,000 live female births that results from loss-of-function mutations in the X-linked gene *MECP2* (methyl-CpG-binding protein 2), encoding a multifunctional protein that regulates gene expression and chromatin architecture by interacting with methylated nucleotides (Amir et al., 1999; Lyst and Bird, 2015). The clinical symptoms currently used as diagnostic criteria for RTT include an early neurologic regression, occurring after a period of typical development, that severely affects motor, cognitive, and communication skills (Smeets et al., 2012). Among the most severe clinical signs associated with RTT are the partial or complete loss of acquired purposeful hand skills and spoken language, the development of gait abnormalities (diagnosed as dyspraxia or apraxia), stereotypic hand movements, and the progressive deterioration of motor abilities (Temudo et al., 2008; Neul et al., 2010; Downs et al., 2016) until walking is completely prevented in many RTT individuals. Although sensory-motor deficits are considered among the most debilitating symptoms of RTT individuals, experimental studies providing insights into the underlying pathophysiological mechanisms are needed.

To investigate the neuropathology produced by the lack of *Mecp2*, several models have been generated including the *Mecp2* ^tm1.1Jae^-null model, used in this study, which present early onset of the neurologic symptoms characteristic of RTT (Chen et al., 2001). On the other hand, due to random X-chromosome inactivation, female *Mecp2* heterozygous (Het) mice recapitulate the cellular mosaicism of *Mecp2* expression found in RTT individuals and closely phenocopy the human condition, including the typical regression of acquired behavioral abilities (Katz et al., 2012; Samaco et al., 2013). Although *Mecp2* is ubiquitously expressed, brain-specific deletion of *Mecp2* in mice entirely recapitulates RTT-like phenotypes, suggesting that its function is most critical in brain cells (Chen et al., 2001; Guy et al., 2001). Moreover, recent studies revealed that loss of *Mecp2* in specific neuronal subtypes leads to distinct RTT-like symptoms (Chao et al., 2010; Zhang et al., 2014), with GABAergic interneurons (INs) emerging as major players in RTT pathophysiology, especially parvalbumin-positive (PV^+^) INs (Durand et al., 2012; Tomassy et al., 2014; Krishnan et al., 2015; Banerjee et al., 2016; Krishnan et al., 2017). Indeed, mice with conditional deletion of *Mecp2* in PV^+^ INs develop a wide range of functional and behavioral RTT-like symptoms (He et al., 2014; Ito-Ishida et al., 2015), including atypical sensory-motor learning. PV^+^ IN activity regulates high-order processing in the cortex by generating precisely timed inhibition/disinhibition of output target neurons (Zhang and Sun, 2011; Pi et al., 2013). Importantly, the plasticity of PV^+^ IN responses controls the activity of specific neocortical circuits and contributes to sensory and motor information processing during behavioral learning (Cardin et al., 2009; Isomura et al., 2009; Sachidhanandam et al., 2016). For instance, PV^+^ INs in the motor cortex exhibit cellular and molecular plasticity when a mouse learns a novel sensory-motor task, such as the rotarod test. This plasticity consists of a shift toward reduced levels of PV, identified as “low-PV-network configuration,” that is caused by changes in the ratio of excitatory and inhibitory boutons converging onto PV^+^ INs. Recently, subpopulations of functionally distinct PV cells have been classified based on levels of PV expression (Donato et al., 2013, 2015; Caroni, 2015). Importantly, an altered PV-network configuration interferes with experience-dependent plasticity mechanism in the brain and is associated with defective motor learning (Donato et al., 2013).

Because *Mecp2* mutant mice show severe motor deficits, we hypothesized that lack of MeCP2 may cause an atypical excitatory/inhibitory connectivity balance onto PV^+^ INs which could alter the configuration of PV networks in the sensory-motor cortices affecting behavioral responses. To test this idea, we first analyzed both the expression of PV and the organization of excitatory and inhibitory synaptic inputs converging onto INs in somatosensory and motor cortices of symptomatic male *Mecp2* KO mice. Next, we investigated these properties in presymptomatic *Mecp2* KO mice before the appearance of overt pathologic symptoms (De Filippis et al., 2010). We then characterized both amplitude and spatial spread of synaptically induced neuronal depolarizations in acute cortical slices to test the impact of the lack of *Mecp2* on activity before and after symptom appearance. Moreover, we evaluated learning-induced changes of PV expression and synaptic connectivity in the M1 cortex of *Mecp2* KO mice. Finally, we studied the developmental trajectory of PV-network configuration and learning-dependent plasticity in female *Mecp2* heterozygous mice. Our results disclose cellular and molecular alterations affecting PV^+^ INs in the sensory-motor cortices that could be responsible for the deficits in motor performance and learning shown by *Mecp2* mutant mice.

## Materials and Methods

### Animals

All animal procedures were performed in accordance with the European Community Council Directive 2010/63/UE for care and use of experimental animals and were approved by the Italian Ministry of Health (Authorization Nr. 175/2015-PR) and by the Bioethical Committee of the University of Turin. At the University of Alabama at Birmingham (UAB), animals were handled and housed according to the Committee on Laboratory Animal Resources of the US National Institutes of Health, and following protocols reviewed annually by the Institutional Animal Use and Care Committee (IACUC-09021). All efforts were made to minimize animal suffering and to reduce the number of animals used. Experimental male *Mecp2* knockout (*Mecp2* KO), female *Mecp2* heterozygous mice (*Mecp2* Het), and wild-type (WT) mice used for this study were obtained by crossing female *Mecp2* Het mice (MMRRC Cat# 000011-UCD, RRID:MMRRC_000011-UCD; *Mecp2^tm1.1Jae^*) with male WT C57BL/6J mice for one generation, followed by breeding among offspring and maintaining on a mixed background (Chen et al., 2001). At UAB, *Mecp2^tm1.1Jae^* mice were maintained on a pure C57BL/6J background. Age-matched WT littermates were used in all experimental conditions to avoid possible consequences of genetic background unrelated to the *Mecp2* mutation. All analyses were conducted by an investigator blinded to both genotype and training of mice.

### Voltage-sensitive dye (VSD) imaging and extracellular field recordings

For VSD imaging and extracellular recordings, presymptomatic [postnatal day 24 (P24)–P26] and symptomatic (P45–P60) *Mecp2* KO mice and WT littermates were anesthetized with ketamine (100 mg/kg i.p.) and transcardially perfused with ice-cold “cutting” artificial cerebrospinal fluid (aCSF) containing (in mM): 87 NaCl, 2.5 KCl, 1.25 NaH_2_PO_4_, 0.5 CaCl_2_, 7 MgCl_2_, 25 NaHCO_3_, 25 glucose, 75 sucrose saturated with 95% O_2_/5% CO. 300-μm-thick slices from the S1 cortex (bregma –0.70 –1.70 mm) were prepared using a vibratome (Leica VT1200S). Slices were incubated at 32°C for 30 min and subsequently allowed to recover for at least 1 h at room temperature (RT) in recording aCSF containing (in mM): 125 NaCl, 25 NaHCO_3_, 25 glucose, 2.5 KCl, 1.25 NaH_2_PO_4_, 2 CaCl_2_, and 1 MgCl_2_ and were saturated with 95% O_2_/5% CO_2_. After recovery, slices were incubated for 45 min at RT with the voltage-sensitive fluorescent dye RH-414 at 30 µM diluted in recording aCSF. Excess dye was washed for at least 20 min in an immersion chamber perfused (2 mL/min) with recording aCSF at RT. RH-414 (absorption 532 nm, emission 716 nm) was excited with 530 ± 50-nm light from a phosphor-pumped LED engine (Heliophor, 89North), and its filtered emission (580 beam-splitter, 594nm long-pass) imaged with a 10× (0.5 NA) Plan-Neofluar objective (Carl Zeiss) in an inverted microscope (Olympus IX-71), and acquired with a scientific CMOS camera running at 2500 frames per second (128 × 128 pixels; NeuroCMOS-SM128 Red Shirt Imaging). Extracellular field excitatory postsynaptic potentials (fEPSPs) were evoked by stimulation of layer V in somatosensory cortex (S1) with a bipolar electrode using constant current pulses (0.1 ms, 0–120 µA) from an isolated stimulator (ISO-Flex, AMPI), and recording in layer II/III using an Axoclamp-2A amplifier (Molecular Devices). Input-output relationships of VSD signals were obtained using stimulation intensities that evoked fEPSPs of 25%, 50%, 80%, and 100% of maximum responses without a population spike. Data were analyzed using custom written Matlab (Mathworks) scripts. The amplitude of VSD signals is expressed as Δ*F*/*F*, and spatio-temporal spread calculated as percentage of cortical layers I-V showing responses above a threshold of 1.5 × 10^−3^ Δ*F*/*F*. Percentage of cortical area was used to account for reduced cortical thickness observed in *Mecp2* KO mice (Fukuda et al., 2005). VSD signals used to compare spatial spread over time were evoked at stimulation intensities eliciting 80% of the maximum fEPSP amplitude. The number of slices and animals for each group was as follows: 12 slices from 4 WT and 24 slices from 6 *Mecp2* KO mice at P45–P60; 17 slices from 3 WT and 3 *Mecp2* KO mice at P24–P26. The areas under the curve of spatial spread over time were compared with Student’s *t* test, and all other comparisons were performed by two-way ANOVA with Bonferroni’s *post hoc* analysis.

### Motor behavioral test: accelerating rotarod

Two-month-old male *Mecp2* KO mice, 8-mo-old female *Mecp2* Het mice, and age-matched WT littermates were tested for motor learning, using an accelerating rotarod apparatus (Ugo Basile). The mice were randomly assigned to the accelerating rotarod (RR test) or to the activity control test (AC test). Mice were tested for two consecutive days, four trials each day, with a 5-min rest interval between trials. The test started when mice were placed and stable on the rod, routed to running forward while the rod was rotating. Each RR trial lasted for a maximum of 5 min, during which the rod accelerated linearly from 4 to 40 rpm, while in AC test a constant speed of 4 rpm was maintained. The time that it took for each mouse to fall from the rod (latency to fall) was recorded for each trial. If the mouse held on to the rod and rotated 360°, this time was noted, and the time of the second rotation was reported as the time of falling off the rod. For immunofluorescence experiment mice were sacrificed 2 h after the last rotarod session. Data were analyzed with a two-way ANOVA and Bonferroni’s *post hoc* analysis (genotype × trial).

## Immunofluorescence

Animals were anesthetized with an intraperitoneal injection of a mixture of Telazol (Zoletil: 80 mg/kg, Alcyon) and Xylazine (Nerfasin 2%: 10 mg/kg, Alcyon) and transcardially perfused with PBS followed by ice-cold paraformaldehyde [4% in 0.1 m phosphate buffer (PB), pH 7.4]. After perfusion, brains were dissected and kept in the same fixative solution overnight at 4°C. After several washes in PB 0.1 m, the brains were cryoprotected by immersion in 10%, 20%, and 30% sucrose solutions and cut in 30-µm coronal sections with a cryostat. Free-floating cryosections were stored at –20°C in a solution containing 30% ethylene glycol and 25% glycerol in 0.1 m PB (pH 7.2) until used. For free-floating immunostaining, following a blocking step in a PBS solution containing 0.05% Triton X-100 and 10% normal donkey serum (NDS), sections were incubated overnight at RT with the following primary antibodies diluted in PBS with 0.05% Triton X-100 and 3% NDS: rabbit anti-calretinin (Synaptic Systems Cat# 214 102 Lot# RRID:AB_2228331;1:2000), guinea pig anti-calretinin (Synaptic Systems Cat# 214 104 Lot# RRID:AB_10635160; 1:500), mouse anti-parvalbumin (Swant Cat# 235 Lot# RRID:AB_10000343; 1:10000), guinea pig anti-VGLUT1 (Millipore Cat# AB5905 Lot# RRID:AB_2301751; 1:5000), and rabbit anti-VGAT (Synaptic Systems Cat# 131 003 Lot# RRID:AB_887869; 1:1000). Double immunofluorescence was performed with simultaneous addition of the primary antibodies. Sections were washed in PBS (3 × 10 min) and incubated for 1 h at RT with the following fluorescent secondary antibodies at 1/1000 dilution: anti-guinea pig Alexa Fluor 488 (Jackson ImmunoResearch Labs Cat# 706-545-148 Lot# RRID:AB_2340472), anti-rabbit Cy3 (Jackson ImmunoResearch Labs Cat# 711-165-152 Lot# RRID:AB_2307443), anti-mouse Cy3 (Jackson ImmunoResearch Labs Cat# 715-165-151 Lot# RRID:AB_2315777), anti-rabbit Cy5 (Jackson ImmunoResearch Labs Cat# 711-175-152 Lot# RRID:AB_2340607), and anti-guinea pig Cy3 (Jackson ImmunoResearch Labs Cat# 706-165-148 Lot# RRID:AB_2340460). Sections were counterstained with the nuclear dye DAPI (nuclear diamidino-2-phenylindole staining, Sigma). After several PBS rinses, the sections were mounted on glass slides and imaged in a wide-field epifluorescence microscope (Eclipse 800, Nikon) equipped with a CCD camera (Axiocam HRc, Carl Zeiss) or in a confocal microscope (Zeiss LSM-5 Pascal) by using sequential acquisition of separate wavelength channels to avoid fluorescence crosstalk.

### Quantification of excitatory and inhibitory presynaptic boutons

To image VGLUT1- and VGAT-positive presynaptic boutons onto PV- and CR-expressing cells, we used the confocal microscope with a 100× oil-immersion objective (1.40 NA) and the pinhole set at 1.0 Airy unit. Each confocal image was composed of 3–4 optical sections spaced 0.5 µm. A minimum of 10 images per animal acquired from layer II/III of the S1 or M1 cortices were used. Images were processed for background subtraction and smoothing filtering with Imaris software (RRID:SCR_007370; release 4.2, Bitplane). Presynaptic puncta that contacted immunolabeled cell bodies and those in close juxtaposition to immunolabeled dendrites were identified as presynaptic varicosities by visual examination in three orthogonal planes using Imaris. Puncta immunolabeled for VGLUT1 or VGAT were considered to be in close apposition to either PV- or CR-positive somata or dendrites when there were no black pixels between the pre- and postsynaptic structures, as described in Pizzo et al. (2016). The number of immunolabeled presynaptic puncta was counted manually using Imaris, and they were included in the analysis only if present in at least two consecutive optical sections in the 3D *z*-axis confocal image stack. The density of immunopuncta (puncta/µm) was calculated by measuring the length of dendritic segments or the perimeter of cell bodies contacted by the axonal terminals using the measuring tool in ImageJ software (RRID:SCR_003070, National Institutes of Health).

### PV immunoreactivity: cell density and signal intensity

To examine PV immunoreactivity, we acquired images from at least 3 coronal brain sections that included S1 or M1 cortices according to a mouse brain atlas (Franklin and Paxinos, 1997) from *Mecp2* KO and WT mice using an epifluorescence microscope (Eclipse 800, Nikon) with a 10× objective. Digital boxes spanning from the pia surface to the white matter (corpus callosum) were superimposed at matched locations on each coronal section of the cerebral cortex. Layer II/III area was identified based on DAPI staining as a cytoarchitectonic reference and measured using ImageJ. A square frame of 80 × 80 µm was placed in the region of the corpus callosum to measure background values, and the background average was subtracted from each section. The total density of PV-immunolabeled cells and their mean fluorescence intensity, measured as average pixel intensity of gray values (0–255) in each cell body, was quantified automatically using the ImageJ Plugin Nucleus Counter. First, we analyzed cumulative distributions of PV^+^ cell intensity for each group of animals. We next classified PV^+^ cells into four subclasses, based on PV fluorescence intensity for each condition and cortical area, as follows: we calculated lower (*l*) and higher (*h*) PV intensity values for the WT group (i.e., the means of the three lower and three higher values for each WT mice) and the intermediate (*i*) value between *l* and *h* values. These values were next used to determine the range of intensities in which the four subclasses of PV^+^ INs were subdivided as follows: low-PV 0 (gray value) – *l*; medium-low PV *l* – *i*; medium-high PV *i – h*; high-PV *h* – 255 (gray value). Data were expressed as fraction (%) of PV^+^ INs or cells/mm^2^ when total density was statistically different between genotypes. Cumulative frequency distributions data were compared using Mann–Whitney *U* test, and the fraction of PV^+^ neurons were compared using Student’s *t* test or two-way ANOVA.

### Statistical analysis

Averages of multiple measurements are presented as mean ± SEM. For each experiment, *n* values are indicated in the figure legends, where *n* stands for the number of animals unless otherwise indicated. Data were statistically analyzed using unpaired two-tailed Student’s *t* test, two-way ANOVA with Bonferroni’s *post hoc* analysis, Mann–Whitney *U* and Pearson correlation tests as indicated in figure legends and in Materials and Methods subsections. All the statistical analyses were performed using Prism software (Graphpad, RRID:SCR_002798), and probability values lower than 0.05 were considered statistically significant. Statistical test results are included in Table 1. Power analysis of the statistical tests was performed using G*Power 3.1.9.2 (RRID:SCR_013726) or SPSS Statistic 22 (RRID:SCR_002865). The power of the statistical tests is reported in Table 2.

**Table 1.** P values of the indicated statistical comparisons.

Figure	Measurement	Type of test	Comparison	Age	*P* value
Fig. 1*B*	PV cell density	Unpaired *t* test	WT vs. *Mecp2* KO	P56	*t*_(10)_ = 2.82, *p* = 0.018
Fig. 1*C*	PV fluorescence intensity	Unpaired *t* test	WT vs. *Mecp2* KO	P56	*t*_(10)_ = 3.24, *p* = 0.009
Fig. 1*D*	Relative frequency of PV INs	Mann–Whitney *U* test	WT vs. *Mecp2* KO	P56	*p* < 0.001
Fig. 1*E*	Fraction of PV INs	Unpaired *t* test	WT vs. *Mecp2* KO (high PV)	P56	*t*_(10)_ = 3.31, *p* = 0.008
			WT vs. *Mecp2* KO (medium-high PV)	P56	*t*_(10)_ = 1.91, *p* = 0.085
			WT vs. *Mecp2* KO (medium-low PV)	P56	*t*_(10)_ = 1.93, *p* = 0.083
			WT vs. *Mecp2* KO (low PV)	P56	*t*_(10)_ = 1.81, *p* = 0.101
Fig. 1*G*	PV cell density	Unpaired *t* test	WT vs. *Mecp2* KO	P28	*t*_(10)_ = 0.61, *p* = 0.555
Fig. 1*H*	PV fluorescence intensity	Unpaired *t* test	WT vs. *Mecp2* KO	P28	*t*_(10)_ = 3.24, *p* = 0.268
Fig. 1*I*	Relative frequency of PV INs	Mann–Whitney *U* test	WT vs. *Mecp2* KO	P28	*p* = 0.002
Fig. 1*J*	Fraction of PV INs	Unpaired *t* test	WT vs. *Mecp2* KO (high PV)	P28	*t*_(10)_ = 0.32, *p* = 0.756
			WT vs. *Mecp2* KO (medium-high PV)	P28	*t*_(10)_ = 1.92, *p* = 0.083
			WT vs. *Mecp2* KO (medium-low PV)	P28	*t*_(10)_ = 1.29, *p* = 0.228
			WT vs. *Mecp2* KO (low PV)	P28	*t_*(10)*_* = 1.54, *p* = 0.155
Fig. 2*E*	VGLUT1 density on PV dendrites	Unpaired *t* test	WT vs. *Mecp2* KO	P56	*t*_(10)_ = 3.62, *p* = 0.004
Fig. 2*F*	VGAT density on PV dendrites	Unpaired *t* test	WT vs. *Mecp2* KO	P56	*t*_(7)_ = 4.22, *p* = 0.003
Fig. 2*G*	VGLUT1 density on PV soma	Unpaired *t* test	WT vs. *Mecp2* KO	P56	*t*_(10)_ = 4.05, *p* = 0.002
Fig. 2*H*	VGAT density on PV soma	Unpaired *t* test	WT vs. *Mecp2* KO	P56	*t*_(8)_ = 1.97, *p* = 0.08
Fig. 2*M*	VGLUT1 density on CR dendrites	Unpaired *t* test	WT vs. *Mecp2* KO	P56	*t*_(10)_ = 2.50, *p* = 0.03
Fig. 2*N*	VGAT density on CR dendrites	Unpaired *t* test	WT vs. *Mecp2* KO	P56	*t*_(8)_ = 0.19, *p* = 0.85
Fig. 2*O*	VGLUT1 density on CR soma	Unpaired *t* test	WT vs. *Mecp2* KO	P56	*t*_(10)_ = 1.32, *p* = 0.21
Fig. 2*P*	VGAT density on CR soma	Unpaired *t* test	WT vs. *Mecp2* KO	P56	*t*_(8)_= 1.79, *p* = 0.11
Fig. 3*E*	VGLUT1 density on PV dendrites	Unpaired *t* test	WT vs. *Mecp2* KO	P28	*t*_(8)_ = 4.89, *p* = 0.001
Fig. 3*F*	VGAT density on PV dendrites	Unpaired *t* test	WT vs. *Mecp2* KO	P28	*t*_(8)_ = 3.39, *p* = 0.009
Fig. 3*G*	VGLUT1 density on PV soma	Unpaired *t* test	WT vs. *Mecp2* KO	P28	*t*_(14)_ = 0.91, *p* = 0.38
Fig. 3*H*	VGAT density on PV soma	Unpaired *t* test	WT vs. *Mecp2* KO	P28	*t*_(8)_ = 0.24, *p* = 0.81
Fig. 3*M*	VGLUT1 density on CR dendrites	Unpaired *t* test	WT vs. *Mecp2* KO	P28	*t*_(8)_ = 0.24, *p* = 0.82
Fig. 3*N*	VGAT density on CR dendrites	Unpaired *t* test	WT vs. *Mecp2* KO	P28	*t*_(8)_ = 0.29, *p* = 0.78
Fig. 3*O*	VGLUT1 density on CR soma	Unpaired *t* test	WT vs. *Mecp2* KO	P28	*t*_(9)_ = 0.14, *p* = 0.89
Fig. 3*P*	VGAT density on CR soma	Unpaired *t* test	WT vs. *Mecp2* KO	P28	*t*_(8)_ = 0.02, *p* = 0.99
Fig. 4*C*	VSD response (ΔF/F)	Two-way ANOVA	WT vs. *Mecp2* KO	P45-60	Genotype *F*_(1,136)_ = 37.07, *p* < 0.001
Fig. 4*D*	Spatial spread of VSD signal	Two-way ANOVA	WT vs. *Mecp2* KO	P45-60	Genotype *F*_(1,136)_ = 18.26, *p* < 0.001
Fig. 4*E*	Spatial spread of VSD signal	Unpaired *t* test	WT vs. *Mecp2* KO	P45-60	*p* < 0.05
Fig. 4*G*	VSD response (ΔF/F)	Two-way ANOVA	WT vs. *Mecp2* KO	P24-26	Genotype *F*_(1,96)_ = 2.73, *p* = 0.108
Fig. 4*H*	Spatial spread of VSD signal	Two-way ANOVA	WT vs. *Mecp2* KO	P24-26	Genotype *F*_(1,96)_ = 12.44, *p* = 0.001
Fig. 4*I*	Spatial spread of VSD signal	Unpaired *t* test	WT vs. *Mecp2* KO	P24-26	*p* < 0.01
Fig. 5*B*	PV cell density	Unpaired *t* test	WT vs. *Mecp2* KO	P56	*t*_(10)_ = 3.67, *p* = 0.004
Fig. 5*C*	PV fluorescence intensity	Unpaired *t* test	WT vs. *Mecp2* KO	P56	t_(10)_ = 1.74, *p* = 0.112
Fig. 5*D*	Relative frequency of PV INs	Mann–Whitney *U* test	WT vs. *Mecp2* KO	P56	*p* < 0.001
Fig. 5*E*	Fraction of PV INs	Unpaired *t* test	WT vs. *Mecp2* KO (high PV)	P56	*t*_(10)_ = 2.45, *p* = 0.034
			WT vs. *Mecp2* KO (medium-high PV)	P56	*t*_(10)_ = 0.81, *p* = 0.439
			WT vs. *Mecp2* KO (medium-low PV)	P56	*t*_(10)_ = 0.98, *p* = 0.349
			WT vs. *Mecp2* KO (low PV)	P56	*t*_(10)_ = 0.46, *p* = 0.657
Fig. 5*G*	PV cell density	Unpaired *t* test	WT vs. *Mecp2* KO	P28	*t*_(6)_ = 1.43, *p* = 0.202
Fig. 5*H*	PV fluorescence intensity	Unpaired *t* test	WT vs. *Mecp2* KO	P28	*t*_(6)_ = 0.98, *p* = 0.366
Fig. 5*I*	Relative frequency of PV INs	Mann–Whitney *U* test	WT vs. *Mecp2* KO	P28	*p* = 0.023
Fig. 5*J*	Fraction of PV INs	Unpaired *t* test	WT vs. *Mecp2* KO (high PV)	P28	*t*_(6)_ = 0.92, *p* = 0.391
			WT vs. *Mecp2* KO (medium-high PV)	P28	*t*_(6)_ = 0.49, *p* = 0.645
			WT vs. *Mecp2* KO (medium-low PV)	P28	*t*_(6)_ = 2.20, *p* = 0.070
			WT vs. *Mecp2* KO (low PV)	P28	*t*_(6)_ *=* 1.64, *p = 0.152*
Fig. 6*A*	Rotarod task	Two-way ANOVA	Genotype vs. RR	P56	RR *F*_(7,112)_ = 11.62, *p* < 0.0001
					Genotype *F*_(1,16)_ = 11.75, *p* = 0.003
					Interaction *F*_(7,112)_ =1.12, *p* = 0.356
Fig. 6*C*	Relative frequency of PV INs	Mann–Whitney *U* test	WT AC vs. *Mecp2* KO-AC	P56	*p* = 0.0449
			WT AC vs. WT RR	P56	*p* = 0.0015
			KO-AC vs. KO-RR	P56	*p* < 0.001
Fig. 6*D*	Fraction of PV INs	Two-way ANOVA	Genotype vs. RR (medium-high PV)	P56	Interaction *F*_(1,18)_ = 6.19, *p* = 0.023
	Fraction of PV INs	Two-way ANOVA	Genotype vs. RR (high PV)	P56	Interaction *F_*(1,18)*_* = 10.11, *p* = 0.005
					Genotype *F*_(1,18)_ = 20.06, *p* = 0.0003
					RR *F*_(1,18)_ = 5.93, *p* = 0.026
Fig. 6*D*	Correlation between % High PV	Pearson’s *r*	WT and *Mecp2* KO	P56	*r*: –0.653, *p* = 0.006
	and RR performance				
Fig. 7*B*	VGLUT1 density on PV dendrites	Two-way ANOVA	Genotype vs. RR	P56	Genotype *F*_(1,16)_ = 117.91, *p* < 0.001
					RR *F*_(1,16)_ = 11.25, *p* = 0.004
	VGLUT1 density on PV soma	Two-way ANOVA	Genotype vs. RR	P56	Genotype *F*_(1,18)_ = 37.20, *p* < 0.001
					RR *F*_(1,18)_ = 27.33, *p* < 0.001
	VGAT density on PV dendrites	Two-way ANOVA	Genotype vs. RR	P56	Genotype *F*_(1,16)_ = 43.89, *p* < 0.001
					RR *F*_(1,16)_ = 17.78, *p* = 0.0007
Fig. 8*B*	Relative frequency of PV INs	Mann–Whitney *U* test	WT vs. *Mecp2* Het	2 M	*p* = 0.8219

Fig. 8*E*	Relative frequency of PV INs	Mann–Whitney *U* test	WT vs. *Mecp2* Het	4 M	*p* = 0.9798
Fig. 8*F*	Fraction of PV INs	Unpaired *t* test	WT vs. Mecp2 Het (high PV)	4 M	*t*_(10)_ = 1.51, *p* = 0.163
			WT vs. Mecp2 Het (medium-high PV)	4 M	*t*_(10)_ = 3.19, *p* = 0.009
			WT vs. Mecp2 Het (medium-low PV)	4 M	*t*_(10)_ = 1.03, *p* = 0.327
			WT vs. Mecp2 Het (low PV)	4 M	*t*_(10)_ = 0.77, *p* = 0.456
Fig. 8*H*	Relative frequency of PV INs	Mann–Whitney *U* test	WT vs. *Mecp2* Het	8 M	*p* < 0.001
Fig. 8*I*	Fraction of PV INs	Unpaired *t* test	WT vs. Mecp2 Het (high PV)	8 M	*t*_(10)_ = 3.16, *p* = 0.013
			WT vs. Mecp2 Het (medium-high PV)	8 M	*t*_(10)_ = 0.52, *p* = 0.620
			WT vs. Mecp2 Het (medium-low PV)	8 M	*t*_(10)_ = 4.06, *p* = 0.004
			WT vs. Mecp2 Het (low PV)	8 M	*t*_(10)_ = 1.40, *p* = 0.200
Fig. 8*J*	Rotarod task	Two-way ANOVA	Genotype vs. RR	8 M	RR *F*_(7,176)_ = 23.60, *p* < 0.001
					Genotype *F*_(1,176)_ = 42.81, *p* < 0.001
					Interaction *F*_(7,176)_ =0.66, *p* = 0.706
Fig. 8*J*	Correlation between % HIGH PV and RR performance	Pearson’s *r*	WT and *Mecp2* Het	8 M	*r*: –0.481, *p* = 0.024

**Table 2.** Data structure (normal or non-normal distribution), statistical tests, and observed power value of the statistical test

Figure	Data structure	Type of test	Power
Fig. 1*B*	Normal distribution	Unpaired *t* test	0.720
Fig. 1*C*	Normal distribution	Unpaired *t* test	0.757
Fig. 1*E*	Normal distribution	Unpaired *t* test	Low 0.372; medium-low 0.413; medium-high 0.410; high 0.845
Fig. 1*G*	Normal distribution	Unpaired *t* test	0.086
Fig. 1*H*	Normal distribution	Unpaired *t* test	0.186
Fig. 1*J*	Normal distribution	Unpaired *t* test	Low 0.286; medium-low 0.214; medium-high 0.412; high 0.059
Fig. 2*E*	Normal distribution	Unpaired *t* test	0.902
Fig. 2*F*	Normal distribution	Unpaired *t* test	0.823
Fig. 2*G*	Normal distribution	Unpaired *t* test	0.953
Fig. 2*H*	Normal distribution	Unpaired *t* test	0.410
Fig. 2*M*	Normal distribution	Unpaired *t* test	0.616
Fig. 2*N*	Normal distribution	Unpaired *t* test	0.053
Fig. 2*O*	Normal distribution	Unpaired *t* test	0.232
Fig. 2*P*	Normal distribution	Unpaired *t* test	0.352
Fig. 3*E*	Normal distribution	Unpaired *t* test	0.989
Fig. 3*F*	Normal distribution	Unpaired *t* test	0.837
Fig. 3*G*	Normal distribution	Unpaired *t* test	0.136
Fig. 3*H*	Normal distribution	Unpaired *t* test	0.055
Fig. 3*M*	Normal distribution	Unpaired *t* test	0.055
Fig. 3*N*	Normal distribution	Unpaired *t* test	0.057
Fig. 3*O*	Normal distribution	Unpaired *t* test	0.052
Fig. 3*P*	Normal distribution	Unpaired *t* test	0.050
Fig. 4*C*	Normal distribution	Two-way ANOVA with RM	Genotype 0.978
Fig. 4*D*	Normal distribution	Two-way ANOVA with RM	Genotype 0.729
Fig. 4*G*	Normal distribution	Two-way ANOVA with RM	Genotype 1.000
Fig. 4*H*	Normal distribution	Two-way ANOVA with RM	Genotype 0.856
Fig. 5*B*	Normal distribution	Unpaired *t* test	0.910
Fig. 5*C*	Normal distribution	Unpaired *t* test	0.053
Fig. 5*E*	Normal distribution	Unpaired *t* test	Low 0.070; medium-low 0.145; medium-high 0.206; high 0.599
Fig. 5*G*	Normal distribution	Unpaired *t* test	0.228
Fig. 5*H*	Normal distribution	Unpaired *t* test	0.132
Fig. 5*J*	Normal distribution	Unpaired *t* test	Low 0.283; medium-low 0.456; medium-high 0.070; high 0.123
Fig. 6*A*	Normal distribution	Two-way ANOVA with RM	Interaction 0.464; test 0.957; genotype 0.895
Fig. 6*D*	Normal distribution	Two-way ANOVA	(Low) interaction 0.666; test 0.094; genotype 0.228
			(Medium-low) interaction 0.113; test 0.278; genotype 0.919
			(Medium-high) interaction 0.739; test 0.466; genotype 0.524
			(High) interaction 0.914; test 0.721; genotype 0.997
Fig. 6*E*	Normal distribution	Pearson’s *r*	0.829
Fig. 7*B*	Normal distribution	Two-way ANOVA	Interaction 0.996; test 0.940; genotype 1.000
Fig. 7*C*	Normal distribution	Two-way ANOVA	Interaction 0.152; test 0.999; genotype 0.999
Fig. 7*D*	Normal distribution	Two-way ANOVA	Interaction 0.999; test 0.999; genotype 0.993
Fig. 7*E*	Normal distribution	Two-way ANOVA	Interaction 0.209; test 0.723; genotype 0.290
Fig. 8*C*	Normal distribution	Unpaired *t* test	Low 0.062; medium-low 0.536
			Medium-high 0.310; high 0.081
Fig. 8*F*	Normal distribution	Unpaired *t* test	Low 0.108; medium-low 0.154
			Medium-high 0.820; High 0.276
Fig. 8*I*	Normal distribution	Unpaired *t* test	Low 0.234; medium-low 0.942
			Medium-high 0.074; high 0.791
Fig. 8*J*	Normal distribution	Two-way ANOVA with RM	Interaction 0.370; test 1.000; genotype 0.844
Fig. 8*K*	Normal distribution	Pearson’s *r*	0.647

## Results

### Age-dependent increase of the fraction of INs expressing higher levels of PV in S1 cortex of Mecp2 KO mice

**Figure 1.**
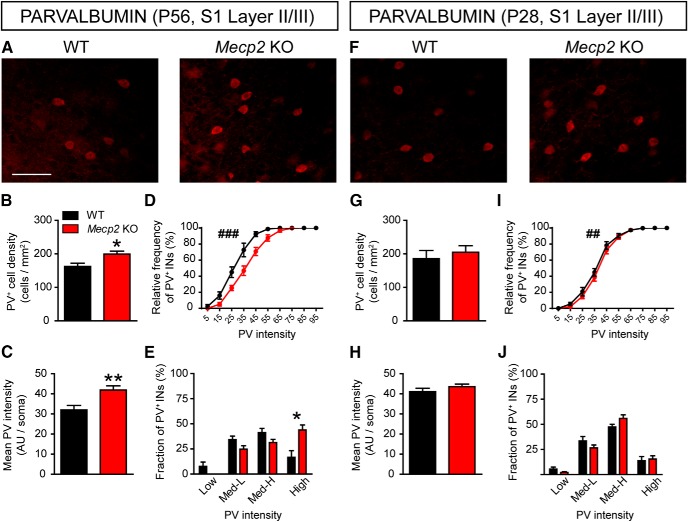
Atypical high-PV expression in the S1 cortex of *Mecp2* KO mice. ***A***, Representative images showing PV labeling in layer II/III of S1 cortex in WT and *Mecp2* KO mice at P56. Histograms showing quantitative analysis of PV^+^ cell density (***B***), PV mean fluorescence intensity (***C***), cumulative (***D***) and binned (***E***) frequency distribution of PV cells intensity in WT and *Mecp2* KO mice at P56. ***F***, Representative images showing PV labeling in layer II/III of S1 cortex in WT and *Mecp2* KO mice at P28. Histograms showing quantitative analysis of PV cell density (***G***), PV mean fluorescence intensity (***H***), cumulative (***I***) and binned (***J***) frequency distribution of PV cells intensity in WT and *Mecp2* KO mice at P28. *n* = 6 mice per genotype. Student’s *t* test: *p < 0.05, **p < 0.01, Mann–Whitney *U* test for ***D***, ***I***: ^##^*p* < 0.01; ^###^*p* < 0.001. Scale bar = 100 μm.

PV^+^ basket cells provide local feed-forward and feedback inhibition onto principal excitatory neurons as well as contributing to the generation of gamma oscillations that are crucial for sensory processing in neocortical circuits (Cardin et al., 2009; Sohal et al., 2009). We therefore evaluated the activation of these cells in the S1 cortex by assessing the levels of PV immunofluorescence, which correlates with both activity and plasticity (Volman et al., 2011; Donato et al., 2013; Fig. 1*A*, *F*). The analysis of S1 cortices from fully symptomatic mice at P56 (De Filippis et al., 2010) confirmed previous studies (Tomassy et al., 2014) showing a significant increase in the density of PV^+^ cells in the upper layers of the cortex in *Mecp2* KO mice compared to WT animals (Fig. 1*B*). Moreover, mean fluorescence intensity (Fig. 1*C*) and cumulative frequency distribution analyses (Fig. 1*D*) indicated that PV intensity showed higher values in *Mecp2* KO mice compared to WT littermates. Using arbitrary criteria to differentiate PV^+^ INs in four subclasses based on fluorescence intensity (see Materials and Methods), we found that in *Mecp2* KO mice there was a robust increase in the percentage of the high-expressing fraction compared to WT animals (Fig. 1*E*). These results were confirmed by the analysis of the absolute density of cells, showing that *Mecp2* loss affects, exclusively, the high-PV subclass (high-PV, WT: 28.55 cells/mm^2^ ± 12.19, *Mecp2* KO: 87.08 cells/mm^2^ ± 9.50. *t* test *t*_(10)_ = 3.79, *p* = 0.004; *n* = 6).

In contrast, there were no significant differences between P28 presymptomatic *Mecp2* KO mice and WT littermates in either the density of PV^+^ INs in layer II/III of the cortex (Fig. 1*G*) or the mean fluorescence intensity of PV (Fig. 1*H*). In addition, while cumulative frequency distribution analysis showed a very modest, although statistically significant, rightward PV intensity shift in mutant mice compared to WT (Fig. 1*I*), the analysis of PV^+^ IN subclasses revealed no significant differences between genotypes at P28 (Fig. 1*J*). Taken together, these results suggest that PV network is shifted toward a high levels of PV expression in the S1 cortex of *Mecp2* KO mice, an alteration that appears when the pathologic signs are established.

### Early-onset supernumerary excitatory connectivity onto PV^+^ INs in Mecp2 KO mice

**Figure 2.**
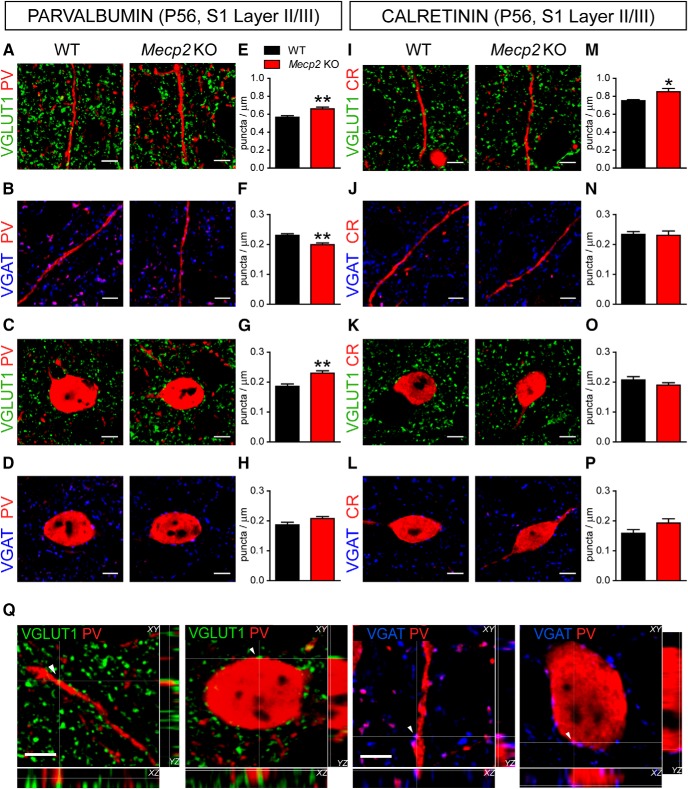
Distribution of excitatory and inhibitory presynaptic terminals onto PV^+^ and CR^+^ INs in P56 *Mecp2* KO mice. Representative confocal images of VGLUT1^+^ (green: ***A***, ***C***) and VGAT^+^ (blue: ***B***, ***D***) puncta corresponding to excitatory and inhibitory presynaptic terminals, respectively, apposed to dendrites (top) and somata (bottom) of PV^+^ INs in layer II/III of S1 cortex in P56 WT and *Mecp2* KO mice. Histograms showing quantitative analysis in WT and *Mecp2* KO mice of VGLUT1^+^ and VGAT^+^ puncta density contacting either dendrites (***E***, ***F***) or somata (***G***, ***H***), respectively, of PV^+^ INs. Confocal images showing VGLUT1^+^ (green: ***I***, ***K***) and VGAT^+^ (blue: ***J***, ***L***) puncta contacting dendrites (top) and somata (bottom) of CR^+^ INs in layer II/III of S1 cortex in WT and *Mecp2* KO mice. Histograms showing quantitative analysis in WT and *Mecp2* KO mice of VGLUT1^+^ and VGAT^+^ puncta density contacting either dendrites (***M***, ***N***) or somata (***O***, ***P***), respectively, of CR^+^ INs. PV: VGLUT1 *n* = 6 mice per genotype; VGAT dendrites *n* = 5 WT and 4 *Mecp2* KO mice per genotype; VGAT soma *n* = 5 mice per genotype; CR: VGLUT1 *n* = 6 mice and VGAT *n* = 5 mice per genotype. Student’s *t* test: *p < 0.05; **p < 0.01. ***Q***, Representative 3D projections in three image planes showing excitatory VGLUT1^+^ (green) and inhibitory VGAT^+^ (blue) synaptic terminals contacting PV^+^ cell bodies and dendrites (red). Arrowheads point to selected VGLUT1^+^ and VGAT^+^ puncta apposed to dendrites or somata of PV^+^ interneurons at the intersection of the XY cross. Note the lack of black pixels between the presynaptic puncta and the postsynaptic structures. Scale bars = 5 μm.

To assess possible mechanisms underlying the altered PV-configuration in *Mecp2* mutant mice, we analyzed excitatory/inhibitory (E/I) input connectivity of identified PV^+^ INs inspecting S1 sections using double immunofluorescence and confocal microscopy. Excitatory presynaptic terminals were identified with antibodies for the vesicular glutamate transporter (VGLUT1), while inhibitory presynaptic terminals were identified using antibodies for vesicular GABA transporter (VGAT; Fig. 2*Q*).

In symptomatic *Mecp2* KO mice at P56, the density of VGLUT1^+^ puncta on PV^+^ dendrites and somata in layer II/III was significantly higher compared to age-matched WT mice (Fig. 2*A*, *C*, *E*, *G*). To exclude that higher density of VGLUT1^+^ puncta is secondary to the smaller size of PV^+^ INs shown by these mutants (Tomassy et al., 2014), we compared the mean number of puncta decorating individual somata in each genotype. This analysis showed that *Mecp2* KO mice are contacted by a significant higher number of VGLUT1^+^ puncta per PV^+^ cell body than WT mice (WT 7.58 ± 0.39 boutons/soma, Mecp2 KO 8.63 ± 0.18; *t* test *t*(10) = 2.396, *p* = 0.037; *n* = 6). Moreover, we found that the density of VGAT^+^ puncta on PV^+^ dendrites was significantly lower in *Mecp2* KO mice compared to WT mice (Fig. 2*B*, *F*), while somata inhibitory innervation was not affected (Fig. 2*D*, *H*). *Mecp2* KO mice at P56 showed higher density of excitatory VGLUT1^+^ presynaptic terminals on CR^+^ dendrites (Fig. 2*I*, *M*), but not on the somata (Fig. 2*K, O*). In contrast, the densities of VGAT^+^ terminals on both dendrites and somata of CR^+^ INs were not statistically different between genotypes (Fig. 2*J*, *N*, *L*, *P*). These observations indicate that in symptomatic *Mecp2* KO there is a shift toward increased excitatory synaptic innervation in both PV^+^ and CR^+^ INs, with PV^+^ INs showing a higher extent of altered E/I connectivity consistent with higher PV expression in these cells (Donato et al., 2013).

**Figure 3.**
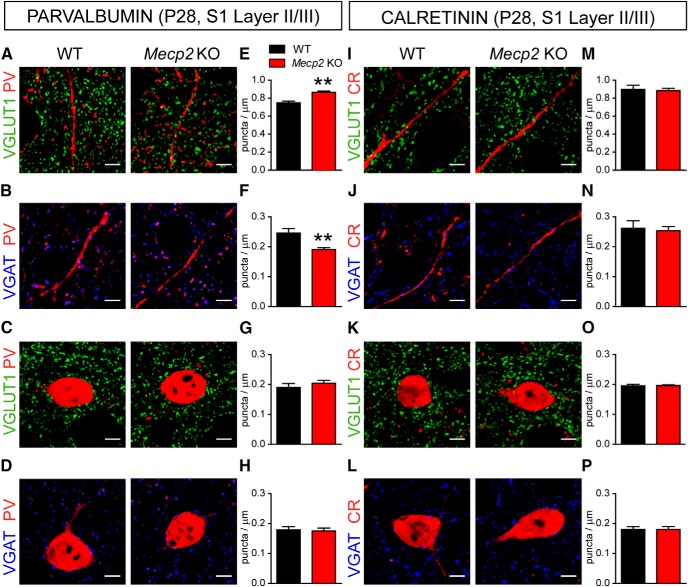
Distribution of excitatory and inhibitory presynaptic terminals onto PV^+^ INs in P28 *Mecp2* KO mice. Representative confocal images of excitatory, VGLUT1^+^ (green: ***A***, ***C***) and inhibitory, VGAT^+^ (blue: ***B***, ***D***) synaptic terminals contacting PV^+^ (red) dendrites (top) and somata (bottom) in layer II/III of S1 cortex in P28 WT and *Mecp2* KO mice. Histograms showing quantitative analysis in WT and *Mecp2* KO mice of VGLUT1^+^ and VGAT^+^ puncta density contacting either dendrites (***E***, ***F***) or somata (***G***, ***H***), respectively, of PV^+^ INs. Representative confocal images of VGLUT1^+^ (green: ***I***, ***K***) and VGAT^+^ (blue: ***J***, ***L***) puncta contacting CR^+^ (red) dendrites (top) and somata (bottom) in layer II/III of S1 cortex in P28 *Mecp2* KO mice and WT littermates. Histograms showing quantitative analysis in WT and *Mecp2* KO mice of VGLUT1^+^ and VGAT^+^ puncta density contacting either dendrites (***M***, ***N***) or somata (***O***, ***P***), respectively, of CR^+^ INs. PV: GLUT1 dendrites *n* = 5 mice per genotype; GLUT1 soma *n* = 8 mice per genotype; VGAT *n* = 5 mice per genotype; CR: VGLUT1 and VGAT *n* = 5 mice per genotype. Student’s *t* test: **p < 0.01. Scale bars = 5 μm.

In presymptomatic *Mecp2* KO mice, the density of VGLUT1-labeled puncta decorating PV^+^ dendrites in layer II/III was significantly higher compared to age-matched WT mice (Fig. 3*A*, *E*), while the number of excitatory inputs on the somata was not affected (Fig. 3*C*, *G*). In contrast, the density of VGAT^+^ puncta on PV^+^ dendrites was significantly lower in *Mecp2* KO mice (Fig. 3*B*, *F*), while inhibitory innervation on PV^+^ somata was unaffected (Fig. 3*D*, *H*). Interestingly, the density of VGLUT1^+^ and VGAT^+^ puncta onto both CR^+^ soma and dendrites in the S1 cortex were comparable between *Mecp2* KO and WT mice at P28 (Fig. 3*I*–*P*). These data indicate that selective alterations in E/I input balance of INs in S1 cortex of presymptomatic *Mecp2* KO animals are specific for the subpopulation of PV^+^ INs and precede the shift toward high-PV expression in this cortical network.

### Early-onset network activity reduction in S1 cortical slices of *Mecp2* KO mice

**Figure 4.**
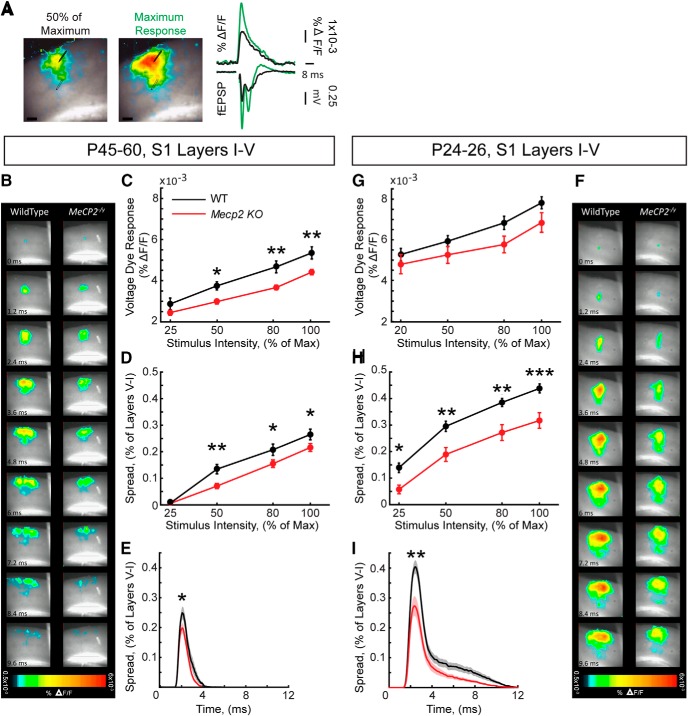
Smaller amplitude and spatial spread of synaptically induced neuronal depolarizations in layer II/III of S1 cortex in presymptomatic and symptomatic *Mecp2* KO mice. ***A***, Representative example (left) of a VSD-stained S1 slice with superimposed evoked VSD signals expressed as Δ*F*/*F*, and displayed in a pseudo-color scale (warmer colors represent larger VSD amplitudes). Representative examples (right) of fEPSPs and VSD Δ*F*/*F* traces at lower (50% maximum response) and higher (maximum response) stimulation intensities. ***B***, ***F***, Frames of representative time-lapse movies of VSD-stained slices during a single fEPSP in symptomatic (***B***) and presymptomatic (***F***) mice. ***C***, ***G***, Input-output relationship between afferent stimulus intensity and the amplitude of VSD signals expressed as % Δ*F*/*F* in symptomatic (***C***) and presymptomatic (***G***) mice. ***D***, ***H***, Input-output relationship between afferent stimulus intensity and the spatial spread of signal through cortical layers I–V in symptomatic (***D***) and presymptomatic (***H***) mice. ***E***, ***I***, Spatio-temporal spread of VSD signals at maximum response stimulation in symptomatic (***E***) and presymptomatic (***I***) mice. Solid lines represent the mean; shaded areas represent the standard error of the mean. *n* = 12 slices from 4 WT mice; *n* = 24 slices from 6 *Mecp2* KO mice at P45-P50; *n* = 17 slices from 3 WT and *Mecp2* KO mice at P24–P26. Two-way ANOVA and Bonferroni posthoc tests for ***C***, ***D***, ***G***, ***H*** and *t* test of area under the curve for ***E***, ***I***. *p < 0.05, **p < 0.01, ***p < 0.001. Scale bars = 100 µm.

To assess the consequences of increased weight of excitation reaching INs in *Mecp2* KO mice on S1 cortex activity, we measured voltage-sensitive dye (VSD) signals evoked by stimulation of intracortical afferents in acute S1 slices from symptomatic (P45–P60) and presymptomatic (P24–P26) *Mecp2* KO and age-matched WT mice. The amplitude of VSD signals (Δ*F*/*F*) evoked in layer II/III by afferent stimulation in layer V was proportional to the amplitude of fEPSPs at different afferent stimulation intensities and followed the same kinetic profile (Fig. 4*A*). The amplitude of VSD signals were significantly smaller in S1 slices from symptomatic *Mecp2* KO mice compared to those from WT mice at all stimulus intensities except the lowest (Fig. 4*C*). In addition, the spatial spread and spatio-temporal profiles of VSD signals through layer I–V was significantly smaller in *Mecp2* KO slices than in WT slices, also at all stimulus intensities except the lowest (Fig. 4*B*, *D, E*). When we analyzed presymptomatic *Mecp2* KO mice, we found a trend toward decreased maximum amplitudes of VSD signals in S1 slices from mutant mice compared with WT slices which did not reach statistical significance (Fig. 4*G*). Moreover, as in older mice, the spatial spread and spatio-temporal profile of VSD signals through layer I–V was significantly smaller in mutant slices than in WT controls at all stimulus intensities (Fig. 4*F*, *H, I*). These results demonstrate that, consistent with an E/I imbalance favoring synaptic inhibition, the S1 cortex of *Mecp2* KO mice is hypoactive in response to layer V stimulation before overt pathologic phenotypes.

### MeCP2 loss disrupts both basal and experience-dependent changes of PV expression in M1 cortex of behaviorally impaired *Mecp2* KO mice

**Figure 5.**
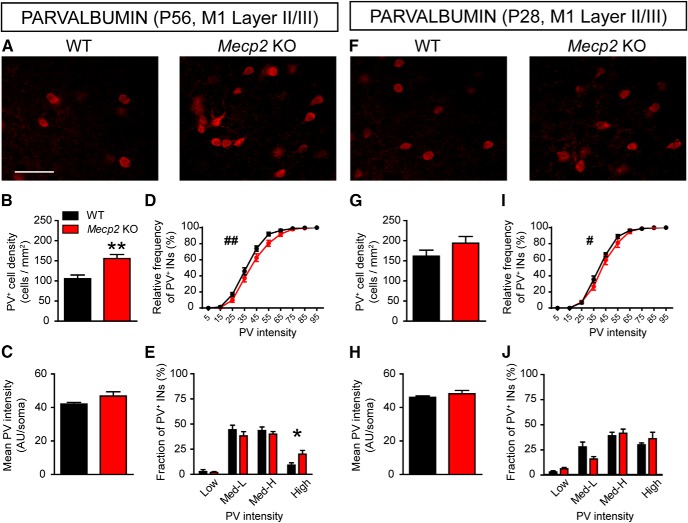
Atypical high-PV-network configuration in the M1 cortex of *Mecp2* KO mice. ***A***, Representative images showing PV expression in layer II/III of M1 cortex in both WT and *Mecp2* KO mice at P56. Histograms showing quantitative analysis of PV^+^ cell density at P56 (***B***), PV mean fluorescence intensity (***C***), cumulative (***D***) and binned (***E***) frequency distribution of PV cells intensity in WT and *Mecp2* KO mice. ***F***, Representative images showing PV expression in layer II/III of M1 cortex in both WT and *Mecp2* KO mice at P28. Histograms showing quantitative analysis of PV cell density (***G***), PV mean fluorescence intensity (***H***), cumulative (***I***) and binned (***J***) frequency distribution of PV cells intensity in WT and *Mecp2* KO mice at P28. *n* = 6 mice per genotype at P56 and *n* = 4 mice per genotype at P28. Student’s *t* test: *p < 0.05, **p < 0.01, Mann–Whitney *U* test for ***D***, ***I***: ^#^*p* < 0.05; ^##^*p* < 0.01. Scale bars = 100 μm.

Although structural alterations affecting pyramidal excitatory neurons in the M1 cortex of both RTT individuals and *Mecp2* KO mice were reported (Armstrong et al., 1998; Belichenko et al., 2009), whether the organization of PV^+^ INs network in this area is affected it is still unknown. Similar to what we observed in S1, P56 *Mecp2* KO mice (Fig. 5*A*) show a significant higher density of PV^+^ INs in layer II/III of the M1 cortex than WT mice (Fig. 5*B*). Moreover, while mean fluorescence intensity of PV was unaffected in M1 of symptomatic mutants (Fig. 5*C*), cumulative frequency distribution analysis showed a shift toward higher PV levels in PV^+^ neurons (Fig. 5*D*), a finding that was corroborated by the significant increase of the percentage of the high-PV fraction compared with WT animals (Fig. 5*E*), which is consistent with our observations in the S1 cortex. These results were confirmed by the analysis of the absolute density of cells, showing that *Mecp2* loss affects, exclusively, the high-PV subclass in M1 cortex (high-PV, WT: 9.98 cells/mm^2^ ± 2.86, *Mecp2* KO: 30.44 cells/mm^2^ ± 5.56. *t* test *t*_(10)_ = 3.27, *p* = 0.008; *n* = 6).

As in S1 cortex, in M1 of P28 mice (Fig. 5*F*) neither the density of PV^+^ INs (Fig. 5*G*) nor the mean fluorescence intensity (Fig. 5*H*) were different between genotypes. Whereas cumulative frequency distribution analysis showed a very modest, although statistically significant, rightward PV intensity shift in mutant mice compared with WT (Fig. 5*I*), the distribution of PV^+^ INs subclasses in M1 revealed no differences between genotypes (Fig. 5*J*). These results indicate that the progression of the pathology in *Mecp2* KO mice is associated with an increased number of cortical INs expressing higher levels of PV.

**Figure 6.**
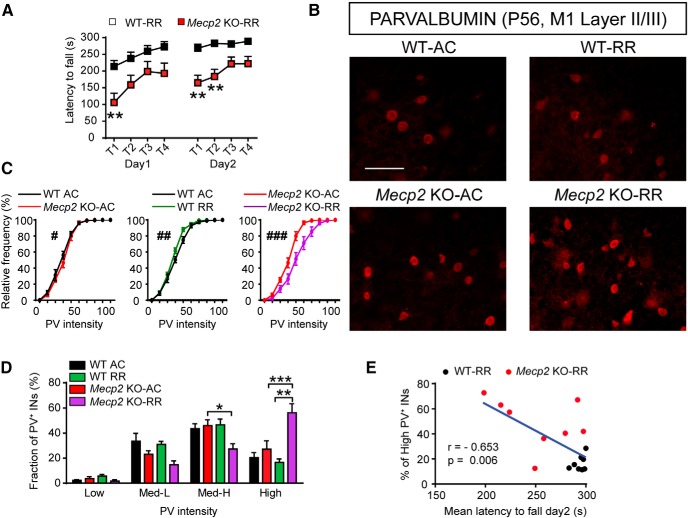
Motor learning–induced plasticity of PV network is impaired in symptomatic *Mecp2* KO mice. ***A***, Latency to fall (seconds) from an accelerating rotating rod in P56 *Mecp2* KO mice and WT littermates. Graphs show data of first and last trials/d (T1–4), for two consecutive days (day 1–2). ***B***, Representative images of PV immunofluorescence in layer II/III INs of the M1 cortex in both WT and *Mecp2* KO P56 mice after AC or RR tasks. Cumulative (***C***) and binned (***D***) frequency distribution of PV cells intensity in layer II/III of M1 cortex, in *Mecp2* KO mice and WT littermates after AC or RR tasks. (***E***) Correlation analysis between the mean latency to fall (seconds) from the rod on day 2 and the fraction of high PV^+^ INs in *Mecp2* KO mice and WT littermates. *n* = 6 WT-AC mice, 5 *Mecp2* KO-AC mice, 6 WT-RR mice, and 5 *Mecp2* KO-RR mice for ***C*** and ***D***. *n* = 9 WT-RR mice and 9 *Mecp2* KO-RR mice for ***A*** and ***E***. Two-way ANOVA and Bonferroni posthoc tests for ***A*** and ***D***: *p < 0.05, **p < 0.01, ***p < 0.001; Mann–Whitney *U* test for ***C***: ^#^*p* < 0.05, ^##^*p* < 0.01, ^###^*p* < 0.001; Pearson’s *r* for ***E***. Scale bar = 100 μm.

It has been previously proposed by Donato et al. (2013) that transitions among different levels of PV expression in M1 represent a molecular signature of PV^+^ IN plasticity reflecting motor learning. To evaluate both motor performance and learning-dependent plasticity of PV^+^ INs in *Mecp2* KO mice, we trained P56 mice on the accelerating rotarod task (RR) for 2 days (8 trials) while activity control (AC) mice ran at a constant speed across trials. Although rotarod performance improved in consecutive trials in both genotypes, P56 *Mecp2* KO mice spent significantly less time on the rod than WT animals (Fig. 6*A*) as previously reported (Stearns et al., 2007). Interestingly, the intensity of PV expression shown by AC mice was only subtly different between genotypes in M1 cortex (Fig. 6*B*) as indicated by cumulative frequency distribution analysis (Fig. 6*C*), probably because AC test may have modified PV expression in M1 reducing the differences between genotypes. Intriguingly, while WT-RR mice exhibited a modest leftward shift toward lower PV intensity level, PV^+^ INs in *Mecp2* KO-RR mice showed a robust rightward shift response (Fig. 6*C*). Moreover, subclasses analysis indicated that *Mecp2* KO-RR mice show a reduced percentage of medium-high-PV cells compared to *Mecp2* KO-AC and a robust increase of the high-PV fraction compared to both *Mecp2* KO-AC and, importantly, WT-RR mice (Fig. 6*D*). Finally, two-way ANOVA analysis revealed a significant effect of both genotype and RR training specifically for the high-PV subclass (Fig. 6*D*).

Remarkably, we found that a greater fraction of high-PV^+^ INs negatively correlated with the motor performance of RR-trained animals (Fig. 6*E*), suggesting that higher PV expression adversely affects behavioral learning. These data indicate that molecular plasticity of PV^+^ INs induced by motor learning is atypical in behaviorally impaired *Mecp2* KO mice, resulting in an abnormal increase of cells expressing high PV levels in the M1 cortex.

### Atypical structural synaptic plasticity on PV^+^ INs in behaviorally impaired *Mecp2* KO mice

**Figure 7.**
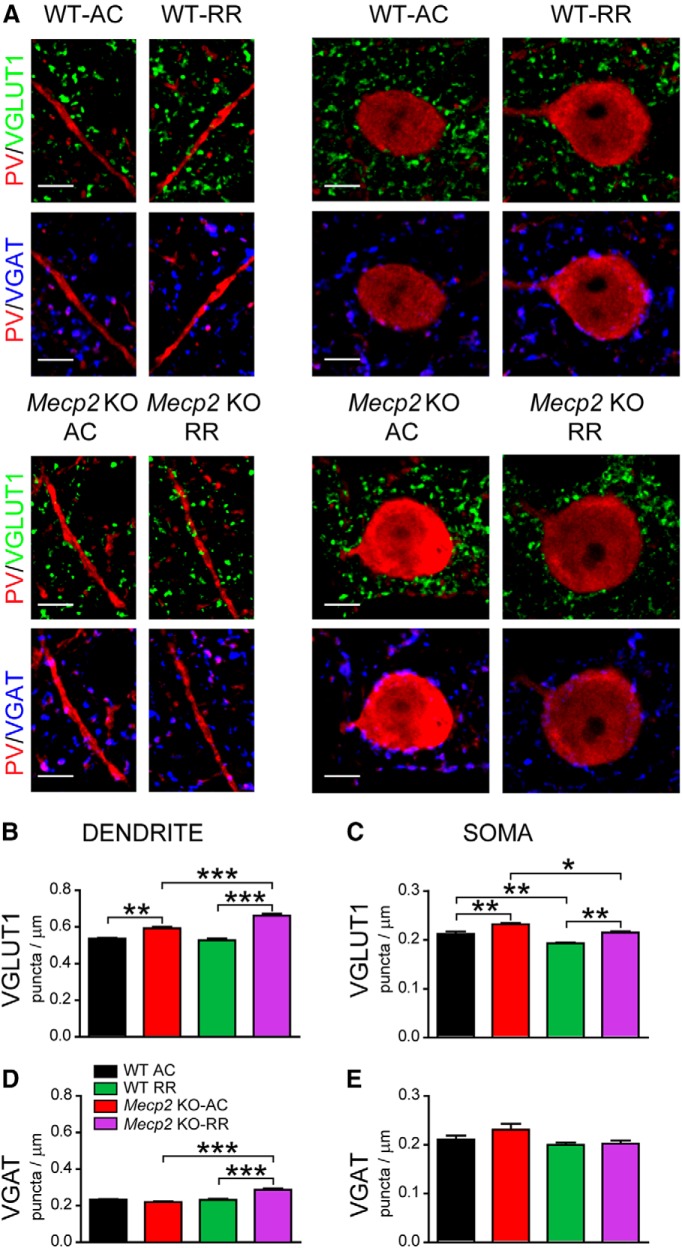
Motor learning produces atypical structural synaptic plasticity of inputs converging on PV^+^ INs in *Mecp2* KO mice. ***A***, Representative confocal images of excitatory VGLUT1^+^ (green) and inhibitory VGAT^+^ (blue) puncta apposed to PV^+^ (red) dendrites and somata in layer II/III of M1 cortex in AC- and RR-trained WT and *Mecp2* KO mice. ***B***, ***C***, Histograms showing quantitative analysis of VGLUT1^+^ puncta density on dendrites (***B***) and somata (***C***) of PV^+^ INs after AC and RR training. ***D***, ***E***, Histograms showing quantitative analysis of VGAT^+^ puncta density on dendrites (***D***) and somata (***E***) of PV^+^ INs after AC and RR training. Dendrites: *n* = 5 WT and 5 *Mecp2* KO mice; somata: *n* = 6 WT and 5 *Mecp2* KO mice. Two-way ANOVA and Bonferroni posthoc tests: *p < 0.05, **p < 0.01, ***p < 0.001. Scale bar = 5 μm.

Changes in PV networks induced by behavioral experience rely on the structural plasticity of both excitatory and inhibitory synapses onto PV^+^ INs (Donato et al., 2013). Therefore, we investigated whether the atypical shift of the distribution of PV-cell subclasses occurring in *Mecp2* KO mice could be produced by aberrant changes of E/I connectivity reaching PV^+^ INs. The density of both excitatory (VGLUT1^+^) and inhibitory (VGAT^+^) presynaptic terminals onto dendrites and somata of layer II/III PV^+^ INs in M1 cortex was quantitatively evaluated on confocal images (Fig. 7*A*). Similar to S1 cortex of untrained animals, the M1 cortex of *Mecp2* KO-AC mice showed a higher density of excitatory boutons decorating both dendritic and soma compartments of PV^+^ INs compared with WT animals (Fig. 7*B*, *C*). Intriguingly, the density of excitatory boutons contacting PV-labeled dendrites was increased in *Mecp2* KO-RR mice (Fig. 7*B*) while there were no changes in WT-RR animals. When we extended this analysis to the somata of PV^+^ cells, we found that RR task produced a significant reduction of VGLUT1^+^ puncta that was similar between genotypes (Fig. 7*C*). In sum, following RR training, *Mecp2* KO mice showed a higher density of putative excitatory synapses contacting PV^+^ INs than WT animals (Fig. 7*B*, *C*).

When we analyzed the density of VGAT^+^ puncta onto PV^+^ INs, we found that there were no differences between genotypes in AC condition (Fig. 7*D*, *E*). Interestingly, we found that the density of VGAT^+^ puncta onto PV^+^ dendrites was increased in *Mecp2* KO-RR mice (Fig. 7*D*) but not in WT-RR animals, resulting in a significant difference in the number of VGAT^+^ terminals between genotypes (Fig. 7*D*). Finally, our analysis showed that RR did not change the number of VGAT^+^ puncta along the somata of PV^+^ INs in both genotypes (Fig. 7*E*). Altogether, these data indicate that behaviorally impaired *Mecp2* KO mice show an atypical activity-dependent structural synaptic plasticity on PV^+^ INs in the M1 cortex that likely produces an aberrant increase of high-PV cell density after motor learning.

### Atypical PV levels in the M1 cortex correlate with both symptom progression and motor coordination disabilities in female *Mecp2* heterozygous mice

**Figure 8.**
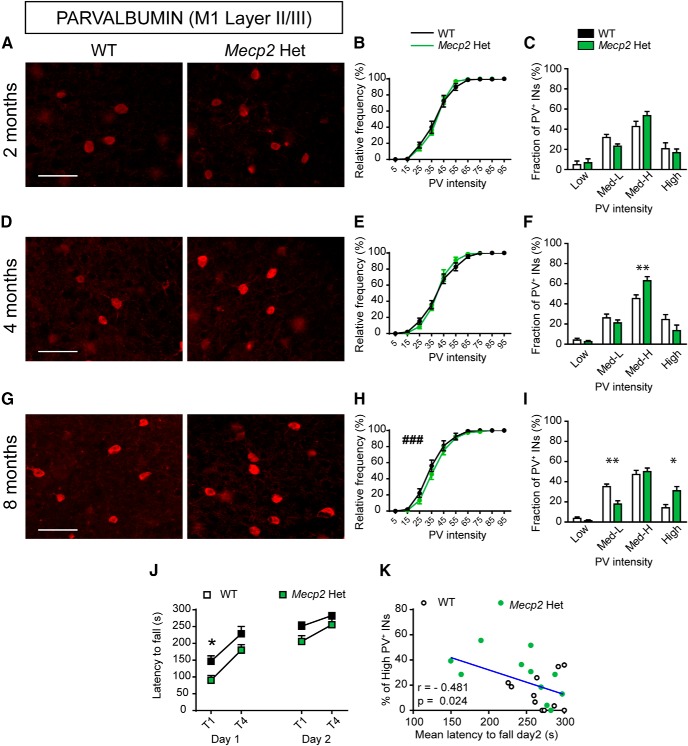
Atypical high-PV expression in the M1 cortex of female *Mecp2* Het mice correlates with motor impairments. Representative images showing PV expression in layer II/III of M1 cortex in both WT and *Mecp2* Het mice at 2 (***A***), 4 (***D***), and 8 (***G***) months of age. Cumulative (***B***, ***E***, ***H***) and binned (***C***, ***F***, ***I***) frequency distribution of PV cells intensity in WT and *Mecp2* Het M1 cortex at 2 (***B***, ***C***), 4 (***E***, ***F***), and 8 (***H***, ***I***) months of age. ***J***, Latency to fall (seconds) from an accelerating rotating rod in 8-mo-old *Mecp2* Het mice and WT littermates. Graphs show data of first and last trials/d (T1–4), for two consecutive days (day 1–2). ***K***, Correlation analysis between the mean latency to fall (seconds) from the rod on day 2 and the fraction of high PV^+^ INs in 8-mo-old *Mecp2* Het and WT females. ***A***–***I***: *n* = 6 WT and 6 *Mecp2* Het mice; ***J***, ***K***: *n* = 11 WT and 11 *Mecp2* Het mice. Mann–Whitney *U* test for ***B***, ***E***, ***H***: ^###^*p* < 0.001; Student’s *t* test for ***C***, ***F***, ***I***: *p < 0.05, **p < 0.01; two-way ANOVA and Bonferroni posthoc tests for ***J***: *p < 0.05, **p < 0.01; Pearson’s *r* for ***E***. Scale bar = 100 μm.

Finally, we assessed PV expression and PV^+^ cell plasticity in female *Mecp2* Het mice, which exhibit mosaic expression of *Mecp2* that resembles the human condition (Katz et al., 2012). As in RTT individuals, female *Mecp2* Het mice have an apparently typical early development, followed by the emergence of several RTT-like symptoms, such as motor impairments, that worsen during the progression of the disease. We analyzed asymptomatic (2-mo-old), presymptomatic (4-mo-old), and severely symptomatic (8-mo-old) mice (De Filippis et al., 2010) to evaluate whether changes in the configuration of PV networks in M1 cortex correlate with symptom appearance (Fig. 8*A*, *D*, *G*). The cumulative frequency distribution analysis revealed a very subtle, although significant, increase of PV intensity in symptomatic *Mecp2* Het mice compared with WT mice, while younger mutants were unaffected (Fig. 8*B*, *E*, *H*). On the other hand, while the analysis of the percentage of PV-cell subclasses showed no differences between genotypes at 2 mo of age (Fig. 8*C*), we found an increase in the high-PV fraction in presymptomatic *Mecp2* Het mice compared with WT animals (Fig. 8*F*), a change that was maintained in severely symptomatic *Mecp2* Het mice (Fig. 8*I*).

We next tested motor coordination in female *Mecp2* Het mice at 8 mo of age and found that these animals showed a significant impairment compared with WT controls in the rotarod task (Fig. 8*J*). Intriguingly, as for *Mecp2* KO mice, a higher fraction of the high-PV cell INs subclass negatively correlated with motor performance of RR-female animals (Fig. 8*K*). Taken together, our analysis indicates that the PV network is affected in *Mecp2* Het female mice and that atypically elevated PV expression is associated with worsened motor coordination shown by these mice.

## Discussion

Dysfunctions of synaptic connectivity and plasticity are thought to be important cellular determinants of RTT and other disorders associated with sensory-motor and intellectual disabilities (Boggio et al., 2010; Zoghbi and Bear, 2012). Revealing the nature of these deficits and identifying the impacted brain areas are crucial to guide the progression and targeting of efficient therapies for these conditions (Shepherd and Katz, 2011; Cortés-Mendoza et al., 2013; Na et al., 2013). Moreover, it is of great importance to characterize the developmental timeline of specific pathologic alterations to establish a putative therapeutic window. In this study, we investigated the putative molecular, synaptic and network determinants underlying motor control and somatosensory processing in *Mecp2* mutant mice. Our results indicate that MeCP2 expression is required for correct synaptic remodeling that regulates the degree of PV^+^ IN network plasticity in response to sensory-motor behavioral learning.

We found for the first time that symptomatic *Mecp2* KO mice show a robust shift of PV^+^ IN networks in both S1 and M1 cortices toward high-PV expression configuration, a condition reflecting atypical plasticity of these INs (Donato et al., 2013). This high-PV configuration was associated with a shift of the E/I input balance toward increased excitatory connectivity that is already present in presymptomatic mutant mice showing only subtle changes of PV expression. When we tested the functional implications of these changes in *Mecp2* KO mice, we found a significant early-onset reduction of stimulus-induced network activity in acute cortical slices consistent with hyperactivated PV inhibitory circuits. Finally, we hypothesized that such altered state of activity and connectivity could impair synaptic plasticity in cortical circuits relevant for motor learning. In support of this hypothesis, we found that lack of MeCP2 disrupts activity-induced pattern of both PV-expression and E/I input rearrangements in PV^+^ INs of the M1 cortex, processes that are associated with the encoding of new motor experiences in this area (Donato et al., 2013). That atypical PV-level adjustment is likely to underlie motor impairments in RTT is strongly supported by our results showing that the larger number of high-PV^+^ INs that we found in M1 cortex of both male *Mecp2* KO and female *Mecp2* Het mice negatively correlates with behavioral performance.

Our study establishes that in *Mecp2* KO mice there is an increase of PV expression in both S1 and M1 cortices, similar to what was previously reported in other neocortical areas (Durand et al., 2012; Patrizi et al., 2015; Krishnan et al., 2015, 2017), further highlighting the role of PV regulation in the progression of RTT pathology. Here, we show for the first time that in layer II/III of both S1 and M1 cortices there is an increase in the density of excitatory presynaptic puncta onto dendrites of PV^+^ INs in P28 and P56 *Mecp2* KO mice compared with WT littermates. Thus, our data indicate that *Mecp2* loss enhances excitatory synaptogenesis on PV^+^ cells, which begins during the period of apparent typical development preceding overt pathologic symptoms, that likely results in higher PV expression. Interestingly, in symptomatic *Mecp2* KO mice there is a further aberrant remodeling of putative excitatory synapses occurring at the somata of PV^+^ cells after synaptogenesis is completed that may represent a primary cause of the aggravation of RTT symptoms. Moreover, our data indicate that the increased density of excitatory terminals onto PV^+^ INs is not determined as a secondary consequence of the smaller cell body area shown by mutant mice (see Tomassy et al., 2014), but instead reflects aberrant excitatory synapse formation and/or maintenance on PV interneurons lacking MeCP2.

In line with this idea, we found that the amplitude and spatio-temporal spread of neuronal depolarizations evoked in layer II/III were significantly smaller in both presymptomatic and symptomatic *Mecp2* KO mice. Because our data suggest that supernumerary PV^+^ INs (see also Tomassy et al., 2014) in layer II/III of *Mecp2* KO mice are hyperactive, it is feasible to suppose that excessive inhibitory drive tampers with both network responses and somatosensory stimuli processing in S1. Although our current results are consistent with observations in V1 cortex of *Mecp2* KO mice (Durand et al., 2012), in this previous study the reduction of VSD propagation response was recognized as stronger PV^+^ cells-to-layer IV pyramidal neuron connectivity. As an alternative, we here suggest that, as a result of excessive excitatory inputs, lack of *Mecp2* leads to atypical hyperactivation of PV^+^ Ins, resulting in decreased cortical network activity. Because these two mechanisms are not mutually exclusive, and since it has been recently proposed that the effectiveness of GABA as an inhibitory transmitter may be altered in *Mecp2* KO mice (Banerjee et al., 2016), it will be necessary to further extend these studies to dissect out the mechanisms leading to atypical cortical activity in the absence of *Mecp2*.

Based on our findings, we propose a dual role of MeCP2 in regulating PV^+^ IN connectivity in both S1 and M1: early, during the developmental processes that shape neural circuits; and late, when synaptic remodeling is mainly produced by experience-dependent plasticity. The early synaptic events caused by *Mecp2* loss primarily stem from defects involving the PV^+^ IN subpopulation, because synaptic connectivity on CR^+^ neurons in presymptomatic *Mecp2* KO mice was like that of WT animals. Moreover, our results indicate that the worsening of the pathologic symptoms observed in developing *Mecp2* KO mice are coincidental with the progressive engagement of additional subclasses of INs, leading to a more extensive pathology of cortical circuits, resulting in altered network activity levels, and hindering the ability of those networks to undergo experience-dependent plasticity. Because it has been previously shown that in the neocortex CR^+^ cells mostly target PV^+^ and somatostatin-expressing cells (Gonchar and Burkhalter, 1999), we cannot exclude that an enhanced excitatory input on CR^+^ cells may represent a homeostatic mechanism to compensate for PV^+^ IN hyperfunctionality. Previous studies have shown that inhibitory synapses from different subclasses of INs target PV^+^ cells on distinct subcellular compartments: PV^+^ puncta, and less frequently somatostatin^+^ terminals, are found to preferentially target the dendrites, while somatic inhibitory inputs targeting PV^+^ INs mainly originate from cells expressing vasoactive intestinal peptide (Hioki et al., 2013). Therefore, consistent with previous observation in V1 (Krishnan et al., 2015), our current findings show that inter-PV^+^ cell communication is selectively affected in the S1 cortex of *Mecp2* KO mice starting at P28.

In line with a late role of *Mecp2*, we find that, unlike WT mice, PV networks in M1 cortex of symptomatic *Mecp2* KO mice shifts toward high-PV expression when performing a rotarod learning task. Consistently, we observed an atypical increase of excitatory connectivity on PV^+^ INs in *Mecp2* KO mice after rotarod learning, accompanied by a weak increase of inhibitory inputs. Our study indicates for the first time that PV^+^ cells in *Mecp2* KO mice undergo aberrant molecular and synaptic changes during the performance and learning of a motor task. Activity-dependent fluctuations of PV expression in M1 cortex of WT mice have been previously associated with synaptic remodeling converging onto these cells (Donato et al., 2013). It is important to note that we have not been able to fully recapitulate the effect size on both PV expression and synaptic remodeling in M1 cortex of WT animals after RR, although we report changes that are coherent with this previous study. These discrepancies are likely due to differences in the anatomic approaches, the criteria used to subdivide PV^+^ IN classes, and importantly, mouse strains. Finally, our study is the first to demonstrate that motor learning–induced synaptic plasticity occurs both on dendritic and somatic compartments of PV^+^ INs, and that lack of *Mecp2* disrupts structural changes of excitatory connectivity onto these cells. Interestingly, it is known that MeCP2 mutation affects the expression of Npas4, a transcription factor that regulates the E/I balance onto INs by modulating the density of excitatory puncta (Ebert et al., 2013). Thus, additional experiments will clarify the molecular mechanisms responsible for the activity-dependent remodeling of synaptic inputs onto PV^+^ INs, both in typically developing mice and in *Mecp2* models.

Finally, we found a similar PV-expression phenotype also in M1 cortex of *Mecp2* Het female mice, suggesting that physiologic levels of expression of MeCP2 protein are important for the configuration and plasticity of PV networks and that a partial reduction of MeCP2 expression is sufficient to interfere with this process. Our study indicates that the increment of PV levels occurs when female *Mecp2* Het mice start to show clear symptoms (Samaco et al., 2013), after a period (i.e., between 2 and 4 months) of typical PV-network configuration. Importantly, because we found a negative correlation between the fraction of high-PV cells in M1 cortex and the motor performance in *Mecp2* Het female mice, it is tempting to speculate that a partial inhibition of PV^+^ INs would be beneficial for the motor impairments in RTT.

## Synthesis

Reviewing Editor: Christian Hansel, University of Chicago Decisions are customarily a result of the Reviewing Editor and the peer reviewers coming together and discussing their recommendations until a consensus is reached. When revisions are invited, a fact-based synthesis statement explaining their decision and outlining what is needed to prepare a revision will be listed below. The following reviewer(s) agreed to reveal their identity: Graziella Di Cristo.

In general, the authors have addressed most of the concerns raised. There are additional minor concerns that need to be addressed:   We are not sure how cumulative distributions in Fig.1I, 5I, 6C and 8C can be significant when curves and error bars overlap.  Is the N use for statistical analysis the number of cells or the number of mice? Using number of cells can lead to statistical significance because cell numbers are large, but cells from the same animal are not independent replicates.   Since the authors use only presynaptic markers and not postsynaptic markers, too, as suggested by the reviewers, we would ask the authors to refer to putative synapses or boutons, and not to synapses throughout the text and data discussion.

It is interesting that the authors believe that the higher PV neuron density is secondary to a smaller brain whereas the higher density of VGlut-1 positive puncta is not. This information does not come across in the manuscript. The authors should reference Tomassy and others in this context, and include the analysis of the mean number of presynaptic puncta contacting the somata of PV cells, in the main text and discussion of the paper.
